# Favorable longitudinal change of lung function in patients with asthma-COPD overlap from a COPD cohort

**DOI:** 10.1186/s12931-018-0737-8

**Published:** 2018-03-02

**Authors:** Hye Yun Park, Suh-Young Lee, Danbee Kang, Juhee Cho, Hyun Lee, Seong Yong Lim, Ho Il Yoon, Seung Won Ra, Ki Uk Kim, Yeon-Mok Oh, Don D. Sin, Sang-Do Lee, Yong Bum Park

**Affiliations:** 1 Division of Pulmonary and Critical Care Medicine, Department of Medicine, Samsung Medical Center, Sungkyunkwan University School of Medicine, Seoul, South Korea; 2 Division of Pulmonary, Allergy, and Critical Care Medicine, Department of Internal Medicine, Hallym University Kangdong Sacred Heart Hospital, 150, Seongan-ro, Gangdong-gu, Seoul, 134-701 South Korea; 3 Department of Clinical Research Design and Evaluation, SAIHST, Sungkyunkwan University, Seoul, South Korea; 4 Center for Clinical Epidemiology, Samsung Medical Center, Seoul, South Korea; 5 Department of Epidemiology and Welch Center for Prevention, Epidemiology, and Clinical Research, Johns Hopkins University Bloomberg School of Public Health, Baltimore, MD USA; 6 Division of Pulmonary and Critical Care Medicine, Department of Medicine, Kangbuk Samsung Hospital, Sungkyunkwan University School of Medicine, Seoul, South Korea; 7 Division of Pulmonary and Critical Care Medicine, Department of Internal Medicine, Seoul National University College of Medicine, Seoul National University Bundang Hospital, Seongnam, South Korea; 8 Department of Medicine, Ulsan University Hospital, University of Ulsan College of Medicine, Ulsan, South Korea; 9 Department of Internal Medicine, Pusan National University School of Medicine, Busan, South Korea; 10 Department of Pulmonary and Critical Care Medicine, Clinical Research Center for Chronic Obstructive Airway Diseases, Asan Medical Center, University of Ulsan College of Medicine, Seoul, South Korea; 11 Respiratory Division, Department of Medicine, University of British Columbia, Vancouver, BC Canada; 12 Lung Research Institute of Hallym University College of Medicine, Chuncheon, South Korea

**Keywords:** Asthma, Chronic obstructive pulmonary disease, Lung function

## Abstract

**Background:**

The recognition of asthma-chronic obstructive pulmonary disease (COPD) overlap (ACO) as a distinct phenotype of COPD or asthma has increased. Although ACO has worse clinical features than non-ACO COPD, limited information is available on long-term outcomes of lung function decline for ACO and non-ACO COPD.

**Methods:**

COPD patients with at least 3 years of follow-up were selected from the Korean Obstructive Lung Disease cohort. ACO was defined based on 3 major criteria: 1) airflow limitation in individuals 40 years of age and older, 2) ≥10 pack-years of smoking history, and 3) a history of asthma or bronchodilator response of > 400 mL in forced expiratory volume in 1 s (FEV_1_) at baseline; and at least 1 minor criterion: 1) history of atopy or allergic rhinitis, 2) two separated bronchodilator responses of ≥12% and 200 mL in FEV_1_, or 3) peripheral blood eosinophils ≥300 cells/μL. Lung function decline was compared using a linear mixed effects model for longitudinal data with random intercept and random slope.

**Results:**

Among 239 patients, 47 were diagnosed with ACO (19.7%). During the follow-up period, change in smoking status, use of inhaled corticosteroids (ICS) and long-acting β2-agonists or ICS and at least 2 exacerbations per year were similar between patients with non-ACO COPD and ACO. Over a median follow-up duration of 5.8 years, patients with non-ACO COPD experienced a faster annual decline in pre-bronchodilator FEV_1_ than patients with ACO (− 29.3 ml/year vs. -13.9 ml/year, *P* = 0.042), which was persistent after adjustment for confounders affecting lung function decline.

**Conclusion:**

Patients with ACO showed favorable longitudinal changes in lung function compared to COPD patients over a median follow-up of 5.8 years.

**Electronic supplementary material:**

The online version of this article (10.1186/s12931-018-0737-8) contains supplementary material, which is available to authorized users.

## Background

Recognition of asthma-chronic obstructive pulmonary disease overlap (ACO) as a distinct phenotype of COPD is increasing [1, 2]. The proportion of ACO varies considerably depending on the diagnostic criteria that are applied, but the overall prevalence of asthma-COPD overlap is approximately 15–30% among patients with obstructive airway disease and 2% among adult population samples [3–6].

Diagnostic criteria for ACO with major and minor criteria were initially proposed by a group of experts in Spain [7]. These have been modified more recently [4]. With growing attention to ACO, a joint project of the Global Initiative for Asthma (GINA) and the Global Initiative for Chronic Obstructive Lung Disease (GOLD) provided a clinical description of asthma-COPD overlap (ACO) that is characterized by persistent airflow limitation with several features usually associated with asthma and several features usually associated with COPD. From the list of common features of asthma and COPD, a diagnosis of ACO is suggested if a similar number of features are found for both asthma and COPD [8]. Due to the imprecise and complicated approach to using the proposed ACO criteria, an operational definition of ACO for clinical and epidemiological application has been advocated by expert consensus from a round table discussion [9].

These attempts to define and diagnose ACO are attributed to different health-care burden and treatment implications. Compared with non-ACO COPD, patients with ACO experience more frequent symptoms of dyspnea and exacerbations with hospitalizations, leading to frequent use of healthcare services and higher healthcare costs [10–12]. Accordingly, patients with ACO are considered to have greater disease severity and a worse prognosis than patients with non-ACO COPD. However, some studies have found the opposite with ACO patients having similar or better prognosis than non-ACO COPD patients [4, 13, 14]. Lung function decline is often used to assess disease progression in COPD and the data on the impact of ACO on disease progression as defined by loss of lung function over time is controversial owing to differences in case definitions of ACO and relative inhomogeneity of the study populations [15–17]. Thus, we aimed to investigate lung function decline between ACO and non-ACO COPD by using a standardized (albeit imperfect) definition of ACO from an expert consensus panel [9] in a relatively homogenous sample of largely male COPD patients in Korea.

## Methods

### Study population

Participants in this study were 239 patients with COPD from the Korean Obstructive Lung Disease (KOLD) cohort. Details of the KOLD study were published previously [18]. Recruitment occurred in pulmonary clinics across 16 hospitals in the Republic of Korea from June 2005 to October 2012. Inclusion criteria for the present study were: 1) 40 years of age and older; 2) COPD, as defined by post-bronchodilator forced expiratory volume in 1 s (FEV_1_)/forced vital capacity (FVC) < 0.7 and more than 10 pack-years of smoking history; 3) no history or radiographic evidence of tuberculosis, bronchiectasis or other pulmonary disorders; and 4) at least 3 years follow-up. At each visit, smoking status was surveyed with a series of questions. Patients were categorized as sustained quitters if they were nonsmokers at each visit. Patients who were smokers at each visit were defined as continued smokers and those whose smoking behavior varied were classified as intermittent quitters. The study protocol was approved by the Institutional Review Board of Asan Medical Center (no. 2005–0010). Written informed consent was obtained from all participants.

### Definition of ACO

Among patients with COPD, those who satisfied three major and at least one minor criterion were classified as ACO patients [9]. Major criteria were: 1) post-bronchodilator FEV_1_/FVC < 0.7, 2) at least 10 pack-years of smoking history, and 3) history of asthma or bronchodilator response to salbutamol/albuterol > 400 mL in FEV_1_ at baseline. Minor criteria were: 1) history of atopy or allergic rhinitis, 2) two separated bronchodilator responses to salbutamol/albuterol ≥12% and 200 mL in FEV_1_ during the initial 3-year follow-up period of the cohort, or 3) peripheral blood eosinophils ≥300 cells/uL.

### Pulmonary function test and computed tomography data acquisition

Spirometry was performed according to recommendations of the American Thoracic Society/European Respiratory Society (Vmax 22, Sensor-Medics, Yorba Linda, CA, USA; PFDX, MedGraphics, St. Paul, MN, USA) [19]. Absolute values of FVC and FEV_1_ were obtained, and percentage of the predicted values (% pred) for FEV_1_ and FVC were calculated from equations obtained with a representative Korean sample [20]. Reversibility was defined as post-bronchodilator increase in FEV_1_ of at least 12% and 200 mL from baseline values.

Details of computed tomography data acquisition and analysis from the KOLD study were published previously [21]. Using in-house software, whole lung images were extracted automatically and attenuation coefficient of each pixel was measured and calculated. Emphysema was defined as a percentage of lung attenuation less than 950 Hounsfield units. Percent emphysema was determined for total lung.

### Statistical analysis

The primary objective of this study was assessing longitudinal changes in pre-bronchodilator FEV_1_ (mL). We compared serial changes in pre-bronchodilator FEV_1_ (mL) between patients with non-ACO COPD and ACO using a linear mixed effects model for longitudinal data with random intercepts and random slopes [22]. After enrollment in the KOLD cohort, some patients underwent a 2-week washout period and then received treatment including a 3-month fixed-dose combination inhaler of inhaled corticosteroids (ICS) and long-acting β2-agonists (LABA) [23]. Patients whose condition did not allow cessation of medications did not undergo the washout and maintained original treatment. This decision was made at the discretion of treating physicians. Since patients who underwent 2-week washout and initiated bronchodilators at enrollment often experienced an increase in FEV_1_ during the first 3 months, we used pre-bronchodilator FEV_1_ at 3 months as the baseline. Due to absence of post-bronchodilator FEV_1_ at 3 months, pre-bronchodilator FEV_1_ was evaluated for lung function decline. We applied three models with increasing degrees of adjustment to account for potential confounding factors. Model 1 was adjusted for baseline age, baseline body mass index (BMI) and smoking status during study period. Model 2 was further adjusted for at least 2 exacerbations per a year during study period. In addition, to evaluate potential mediation of the association between presence of ACO and pre-bronchodilator FEV_1_ changes, model 3 was further adjusted for use of ICS/LABA or ICS during study period. The use of ICS/LABA or ICS was defined as ICS/LABA or ICS was prescribed longer than two-thirds of a study period. All reported *P* values were two-sided and the significance level was set at 0.05. Statistical analyses were performed using Stata (version 14; Stata Corp., College Station, TX).

## Results

In this study, 47 patients (19.7%) fulfilled the definition of ACO (Table 1 and Additional file 1: Table S1). Compared with patients with non-ACO COPD, no significant differences were observed in age, sex, smoking history, education status, modified medical research council ≥2, two or more exacerbations in the previous year and other comorbidities except for bronchial asthma between patients with ACO and non-ACO COPD. While baseline FEV_1_ was similar between non-ACO COPD and ACO (1471.7 mL vs. 1545.5 mL, *P* = 0.38; 48.3% pred vs. 49.7% pred, *P* = 0.56), baseline post-bronchodilator FEV_1_ was higher for ACO than non-ACO COPD (1816.2 mL vs. 1618.5 mL, *P* = 0.02; 58.3% pred vs. 53.1% pred, *P* = 0.04). Reversibility was significantly higher for ACO than non-ACO COPD, while emphysema index was significantly higher for non-ACO COPD than ACO (22.1% vs. 17.1%, P = 0.04). For medications, use of long-acting muscarinic antagonists and ICS/LABA or ICS prescription were similar between non-ACO COPD and ACO (Table 2).

**Table 1:** Baseline characteristics of the study population

	Overall (*N* = 239)	Non-ACO COPD (*n* = 192)	ACO (*n* = 47)	*P*-value
Age, years	66.2 (7.4)	66.6 (7.5)	64.7 (6.6)	0.11
Sex				0.39
Female	6 (2.5)	4 (2.1)	2 (4.3)	
Male	233 (97.5)	188 (97.9)	45 (95.7)	
Smoking history (baseline)				0.93
Current	75 (31.4)	60 (31.3)	15 (31.9)	
Ex-smoker	164 (68.6)	132 (68.8)	32 (68.1)	
Pack-years	47.8 (27.3)	48.5 (26.8)	45.0 (29.2)	0.44
Body mass index, kg/m^2^	23.3 (3.2)	23.2 (3.2)	23.9 (3.1)	0.17
Education				0.89
< High school diploma	109 (45.6)	88 (45.8)	21 (44.7)	
≥ High school diploma	130 (54.4)	104 (54.2)	26 (55.3)	
mMRC ≥2	125 (52.3)	100 (52.1)	25 (53.2)	0.89
SGRQ				
Symptom	44.1 (18.0)	43.7 (17.9)	45.8 (18.7)	0.48
Activity	47.5 (22.8)	47.2 (22.9)	48.6 (22.6)	0.70
Impact	21.2 (18.4)	21.1 (18.2)	21.8 (18.4)	0.80
Total	33.1 (17.4)	32.8 (17.4)	33.9 (17.5)	0.71
Previous exacerbation^a^, ≥2	24 (10.0)	18 (9.4)	6 (12.8)	0.49
Comorbidity				
Tuberculosis	47 (19.7)	36 (18.8)	11 (23.4)	0.47
Bronchial asthma	71 (29.7)	32 (16.7)	39 (83.0)	< 0.001
Cardiovascular disease^b^	68 (28.5)	53 (27.6)	15 (31.9)	0.56
Gastrointestinal disease	48 (20.1)	36 (18.8)	12 (25.5)	0.30
Hepatobiliary disease	14 (5.9)	11 (5.7)	3 (6.4)	0.74^c^
Urogenital disease	37 (15.5)	27 (14.1)	10 (21.3)	0.22
Nervous disease	10 (4.2)	9 (4.7)	1 (2.1)	0.69^c^
Endocrine disease	33 (13.8)	26 (13.5)	7 (14.9)	0.81
Kidney disease	2 (0.8)	2 (1.0)	0	> 1.00^c^
Cancer	3 (1.2)	2 (1.0)	1 (2.1)	0.55^c^
Diabetes mellitus	23 (9.6)	18 (9.4)	5 (10.6)	0.79
Hypertension	72 (30.1)	61 (31.8)	11 (23.4)	0.26

Data are presented as number (%) or as mean (SD)

*Abbreviations*: *ACO* asthma-chronic obstructive pulmonary disease overlap, *COPD* chronic obstructive lung disease, *mMRC* modified Medical Research Council, *SD* standard deviation, *SGRQ* St George’s Respiratory Questionnaire, *ICS* inhaled corticosteroids

^a^Medical history of hospital or emergency room visit for treatment within 1 year before enrollment because of one or more of the following: increased shortness of breath, increased sputum volume, increased sputum purulence

^b^included myocardial infarction, heart failure, peripheral vascular disease, cerebrovascular disease

^c^Fisher’s exact

**Table 2:** Baseline characteristics of lung function, emphysema and use of inhalers of the study population

	Overall (*N* = 239)	Non-ACO COPD (*n* = 192)	ACO (*n* = 47)	*P*-value
Pulmonary Function Test				
FEV_1_ (mL)	1486.2 (517.5)	1471.7 (532.4)	1545.5 (451.9)	0.38
FEV_1_, % predicted	48.6 (15.0)	48.3 (15.2)	49.7 (14.0)	0.56
FVC (mL)	3279.2 (811.1)	3255.6 (809.1)	3375.7 (820.8)	0.36
FVC, % predicted	77.9 (17.0)	77.5 (16.9)	79.4 (17.5)	0.51
FEV1/FVC (%)	45.1 (10.2)	44.9 (10.7)	45.8 (7.8)	0.59
Post-bronchodilator FEV_1_ (mL)	1657.4 (539.9)	1618.5 (540.4)	1816.2 (513.2)	0.024
Post bronchodilator FEV_1_, % predicted	54.1 (15.4)	53.1 (15.3)	58.3 (15.4)	0.039
Post bronchodilator FEV_1_ < 50% predicted, n (%)	102 (42.7)	86 (44.8)	16 (34.0)	0.18
Reversibility, n (%)	85 (35.6)	57 (29.7)	28 (59.6)	< 0.01
Emphysema, (%)	21.1 (14.9)	22.1 (14.9)	17.1 (14.7)	0.044
> 5%	193 (80.8)	161 (83.9)	32 (68.1)	0.014
> 10%	163 (68.2)	137 (71.4)	26 (55.3)	0.034
> 15%	133 (55.7)	110 (57.3)	23 (48.9)	0.30
Inhalers				
LAMA, n (%)	79 (33.1)	62 (32.3)	17 (36.2)	0.61
ICS/LABA or ICS, n (%)	98 (40.7)	73 (38.0)	25 (53.2)	0.051

Data are presented as number (%) or as mean (SD)

*Abbreviations*: *FEV*_*1*_ forced expiratory volume in 1 s, *FVC* forced vital capacity, *ACO* asthma-chronic obstructive pulmonary disease overlap, *COPD* chronic obstructive lung disease, *LAMA* long acting muscarinic antagonists, *LABA* long-acting β2-agonists, *ICS* inhaled corticosteroids

**Fig. 1:**
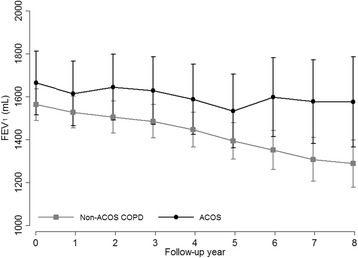
Longitudinal Changes in pre-bronchodilator forced expiratory volume in 1 s (mL) during the follow-up period in non-ACO COPD (*n* = 192) and ACO (*n* = 47). Error bar represents 95% confidence interval. ACO, asthma-chronic obstructive pulmonary disease overlap syndrome; COPD, chronic obstructive lung disease

The median time from baseline spirometry to the last spirometry was 5.8 years (maximum 8.8 years). During follow-up, changes in smoking status, use of ICS/LABA or ICS and at least 2 exacerbations per year were similar between non-ACO COPD and ACO patients (Table 3). During follow-up, patients with non-ACO COPD had a significantly faster annual decline in pre-bronchodilator FEV_1_ than patients with ACO (− 29.26 mL vs. -13.87 mL, *P* = 0.042) (Table 4 and Fig. 1). The results did not substantially change after adjusting for other confounders such as baseline age, baseline BMI, change in smoking status during study period, exacerbations during study period and use of ICS/LABA or ICS during the study period (Table 4).

**Table 3:** Change in smoking status, use of ICS/LABA or ICS and moderate-to-severe exacerbation during the follow-up period

	Overall (*N* = 239)	Non-ACO COPD (*n* = 192)	ACO (*n* = 47)	*P*-value
Smoking Status				0.92
Continued smokers	8 (3.4)	6 (3.1)	2 (4.3)	
Intermittent quitters	96 (40.2)	77 (40.1)	19 (40.4)	
Sustained quitters	135 (56.5)	109 (56.8)	26 (55.3)	
Use of ICS/LABA or ICS^a^	159 (66.5)	125 (65.1)	34 (72.3)	0.35
Exacerbation^b^				
At least 2 incidents per year during follow-up	42 (17.6)	30 (15.6)	12 (25.5)	0.11

Data are presented as number (%)

*Abbreviations*: *ACO* asthma-chronic obstructive pulmonary disease overlap, *COPD* chronic obstructive lung disease, *ICS* inhaled corticosteroids, *LABA* long-acting β2-agonists

^a^Defined as when the ICS/LABA or ICS was prescribed for longer than 2/3 of the study period

^b^Medical history of hospital or emergency room visit for treatment because of one or more of the following: increased shortness of breath, increased sputum volume, and increased sputum purulence, which was assessed at every visit

**Table 4:** Longitudinal changes in annual pre-bronchodilator forced expiratory volume in 1 s (mL) between non-ACO COPD and ACO

	Non-ACO COPD (*n* = 192)	ACO (*n* = 47)	*P* for interaction*
Crude, *mL*	−29.26 (−35.78, −22.75)	−13.87 (− 27.22, − 0.52)	0.042
Model 1, *mL*	− 29.17 (− 35.73, − 22.61)	−13.58 (− 27.01, − 0.14)	0.041
Model 2, *mL*	−29.19 (− 35.74–22.64)	−13.64 (− 27.07, − 0.22)	0.041
Model 3, *mL*	−29.16 (− 35.73, − 22.60)	−13.61 (− 27.06, − 0.17)	0.042

Data are presented as mean (95% confidence interval)

Model 1: Adjusted for baseline age, baseline body mass index and smoking status during the study period; Model 2: Further adjusted for at least 2 exacerbations per a year during study period; Model 3: Further adjusted for use of ICS/LABA or ICS during the study period

*Abbreviations*: *ACO* asthma-chronic obstructive pulmonary disease overlap, *COPD* chronic obstructive lung disease, *ICS* inhaled corticosteroids, *LABA* long-acting β2-agonists

*P value for homogeneity of annual change by group

## Discussion

We found that patients with ACO experienced a slower annual decline in FEV_1_ compared to patients with non-ACO COPD over a median follow-up period of 5.8 years. This finding persisted after adjustment for confounders affecting lung function decline such as change in smoking status, exacerbations and use of ICS/LABA or ICS during the study. Our data are consistent with those of the Hokkaido COPD cohort study [24], which did not include patients with history of asthma but demonstrated a significantly slower annual lung function decline in COPD patients with the presence of three asthma-like features such as blood eosinophilia, reversibility and atopy compared to COPD without asthma-like features. We extended the data of the Hokkaido COPD cohort study by showing that patients with ACO had a slower annual decline in FEV_1_ than patients with non-ACO COPD. Our data also affirmed the finding of a cohort study of obstructive airway diseases with more patients and a longer follow-up period. Previous data showed that there was a significant decline in FEV_1_ over the 4-year period within non-ACO COPD not within ACO, but there were no significant differences in FEV_1_ changes between two groups. These might be explained by the lack of sufficient power to detect differences in lung function decline due to relatively small sample size (*n* = 91) and shorter follow-up period [17].

Two other studies evaluated lung function decline between ACO and non-ACO COPD [15, 16]. One was a population-based study that used a self-reported definition of asthma [15]. This study showed that lung function decline for ACO was affected by the age of asthma recognition. Compared with non-ACO COPD, decline in FEV_1_ for ACO with late asthma onset was significantly higher but decline in FEV_1_ for ACO with early asthma onset was significantly lower. The other study used data from the European Community Respiratory Health Survey (participants aged 20–44 years) and showed that FEV_1_ change in the ACO group was lower than in the COPD group [16]. On further inspection of these two studies, compared with non-ACO COPD, the numbers for initial FEV_1_ < 80% of predicted were significantly higher for ACO with early asthma onset (50% vs. 71%) in the former study and ACO patients (16.4% vs. 33.1%) in latter study. Given that lung function decline accelerates in the initial phase of COPD with preserved FEV_1_ [25], a smaller change in lung function for ACO with early asthma onset in these previous data compared to non-ACO COPD could be attributed to low baseline FEV_1_. However, in our study, baseline pre-bronchodilator FEV_1_ was similar for non-ACO COPD and ACO, and post-bronchodilator FEV_1_ was higher for ACO than non-ACO COPD. In addition, changes in smoking status and numbers of frequent exacerbations during follow-up were similar for ACO and non-ACO COPD, suggesting that favorable lung function decline in ACO compared to non-ACO COPD could be true signal.

The presence of bronchodilator reversibility has been widely used as a criterion in the classification of ACO [4, 7, 17]. Previous studies reported the association between bronchodilator reversibility and lung function decline with conflict results in COPD patients [24, 26–28]. Our data showed a higher rate of bronchodilator reversibility for ACO than non-ACO COPD owing to the inclusion of bronchodilator responses in the case definition of ACO that was used in this study, and the variance of FEV_1_ at each year tended to be wider for ACO than non-ACO COPD (Fig. 1). However, the presence of baseline reversibility in our study did not affect overall lung function decline (data not shown), which was in agreement with the data of the Lung Health Study, Inhaled Steroids in Obstructive Lung Disease in Europe (ISOLDE) study and Hokkaido COPD cohort study [24, 26, 27]. Thus, the effect of bronchodilator reversibility on a slower lung function decline in ACO would not be significant.

When only patients with ACO were analyzed based on ICS/LABA or ICS treatment, a trend towards reduced annual rate of decline in FEV_1_ was found for patients with ACO receiving ICS/LABA or ICS treatment during follow-up compared with those without ICS/LABA or ICS treatment (Additional file 1: Table S2). However, no significant difference was observed in annual rate of FEV_1_ decline for patients with ACO based on use of ICS/LABA or ICS, which might be due to the small sample size. Given that our findings were from retrospective observational studies, a prospective study is needed to validate the effects of ICS on ACO.

Our study has several limitations. First, as the KOLD prospective cohort was not originally designed to compare lung function decline between ACO and non-ACO COPD, our study design and analysis had to be retrospective in nature. Accordingly, we did not compare lung function decline for non-ACO COPD with ACO using different ACO criteria. In particular, ACO criteria proposed by a joint project of GINA and GOLD [8] could not be applied because some data were missing to determine ACO or non-ACO COPD. Second, a history of asthma before 40 years of age could not be used because the age of asthma diagnosis was not investigated in the KOLD cohort. Thus, we modified the ACO criteria with “history of asthma”. Further studies are necessary to investigate lung function decline for ACO based on age of asthma diagnosis in the COPD cohort. Third, despite a long-term follow-up, the sample size might be small to make conclusion. A prospective study with a larger sample size is needed to validate these findings. Finally, 97% of the study population was male. This can be explained by the high prevalence of male smokers in Korea [29]. Thus, our results may be limited to a generalization of female patients.

## Conclusions

We found that the longitudinal change in lung function was lower in patients with ACO than patients with COPD over a median follow-up of 5.8 years. This finding suggested that ACO has favorable long-term outcomes in lung function decline compared with non-ACO COPD in a cohort of Korean patients with COPD.

## Additional file

Additional file 1: **Table S1.** The component distribution of ACO. ** Table S2.** Longitudinal change of annual forced expiratory volume in 1 s (mL) in ACO by use of ICS/LABA or ICS during follow up (*n* = 47). (DOCX 15 kb)

## Abbreviations

ACO: Asthma-chronic obstructive pulmonary disease overlap
BMI: Body mass index
COPD: Chronic obstructive lung disease
FEV_1_: Forced expiratory volume in 1 s
FVC: Forced vital capacity
GINA: Global Initiative for Asthma
GOLD: Global Initiative for Chronic Obstructive Lung Disease
ICS: Inhaled corticosteroids
KOLD: Korean Obstructive Lung Disease
LABA: Long-acting β2-agonists

## References

[1] Barnes PJ (2016). Asthma-COPD overlap. Chest.

[2] Kostikas K, Clemens A, Patalano F (2016). The asthma-COPD overlap syndrome: do we really need another syndrome in the already complex matrix of airway disease?. Int J Chron Obstruct Pulmon Dis..

[3] Alshabanat A, Zafari Z, Albanyan O, Dairi M, FitzGerald JM (2015). Asthma and COPD overlap syndrome (ACOS): a systematic review and meta analysis. PLoS One.

[4] Cosio BG, Soriano JB, Lopez-Campos JL, Calle-Rubio M, Soler-Cataluna JJ, de-Torres JP (2016). Defining the asthma-COPD overlap syndrome in a COPD cohort. Chest.

[5] Tho NV, Park HY, Nakano Y (2016). Asthma-COPD overlap syndrome (ACOS): a diagnostic challenge. Respirology.

[6] Menezes AM, Montes de Oca M, Perez-Padilla R, Nadeau G, Wehrmeister FC, Lopez-Varela MV (2014). Increased risk of exacerbation and hospitalization in subjects with an overlap phenotype: COPD-asthma. Chest.

[7] Soler-Cataluna JJ, Cosio B, Izquierdo JL, Lopez-Campos JL, Marin JM, Aguero R (2012). Consensus document on the overlap phenotype COPD-asthma in COPD. Arch Bronconeumol.

[8] GINA. Global Strategy for Asthma Management and Prevention (2017 update). Available at http://ginasthma.org/2017-gina-report-global-strategy-for-asthma-management-and-prevention/. Assessed 15 Sept 2017.

[9] Sin DD, Miravitlles M, Mannino DM, Soriano JB, Price D, Celli BR (2016). What is asthma-COPD overlap syndrome? Towards a consensus definition from a round table discussion. Eur Respir J.

[10] Hardin M, Silverman EK, Barr RG, Hansel NN, Schroeder JD, Make BJ (2011). The clinical features of the overlap between COPD and asthma. Respir Res.

[11] Miravitlles M, Soriano JB, Ancochea J, Munoz L, Duran-Tauleria E, Sanchez G (2013). Characterisation of the overlap COPD-asthma phenotype. Focus on physical activity and health status. Respir Med.

[12] Sadatsafavi M, Tavakoli H, Kendzerska T, Gershon A, To T, Aaron SD (2016). History of asthma in patients with chronic obstructive pulmonary disease. A comparative study of economic burden. Ann Am Thorac Soc.

[13] Sorino C, Pedone C, Scichilone N (2016). Fifteen-year mortality of patients with asthma-COPD overlap syndrome. Eur J Intern Med.

[14] Yamauchi Y, Yasunaga H, Matsui H, Hasegawa W, Jo T, Takami K (2015). Comparison of in-hospital mortality in patients with COPD, asthma and asthma-COPD overlap exacerbations. Respirology.

[15] Lange P, Colak Y, Ingebrigtsen TS, Vestbo J, Marott JL (2016). Long-term prognosis of asthma, chronic obstructive pulmonary disease, and asthma-chronic obstructive pulmonary disease overlap in the Copenhagen City heart study: a prospective population-based analysis. Lancet Respir Med.

[16] de Marco R, Marcon A, Rossi A, Anto JM, Cerveri I, Gislason T (2015). Asthma, COPD and overlap syndrome: a longitudinal study in young European adults. Eur Respir J.

[17] Fu JJ, Gibson PG, Simpson JL, McDonald VM (2014). Longitudinal changes in clinical outcomes in older patients with asthma, COPD and asthma-COPD overlap syndrome. Respiration.

[18] Park TS, Lee JS, Seo JB, Hong Y, Yoo JW, Kang BJ (2014). Study design and outcomes of Korean obstructive lung disease (KOLD) cohort study. Tuberc Respir Dis (Seoul).

[19] Miller MR, Hankinson J, Brusasco V, Burgos F, Casaburi R, Coates A (2005). Standardisation of spirometry. Eur Respir J.

[20] Choi JK, Paek D, Lee JO (2005). Normal predictive values of spirometry in Korean population. Tuberc Respir Dis.

[21] Oh YM, Jeong BH, Woo SY, Kim SY, Kim H, Lee JH (2015). Association of plasma adipokines with chronic obstructive pulmonary disease severity and progression. Ann Am Thorac Soc..

[22] Fitzmaurice GM, Laird NM, Ware JH (2011). Applied longitudinal analysis.

[23] Lee SY, Park HY, Kim EK, Lim SY, Rhee CK, Hwang YI (2016). Combination therapy of inhaled steroids and long-acting beta2-agonists in asthma-COPD overlap syndrome. Int J Chron Obstruct Pulmon Dis..

[24] Suzuki M, Makita H, Konno S, Shimizu K, Kimura H, Kimura H (2016). Asthma-like features and clinical course of chronic obstructive pulmonary disease. An analysis from the Hokkaido COPD cohort study. Am J Respir Crit Care Med.

[25] Tantucci C, Modina D (2012). Lung function decline in COPD. Int J Chron Obstruct Pulmon Dis.

[26] Calverley PM, Burge PS, Spencer S, Anderson JA, Jones PW (2003). Bronchodilator reversibility testing in chronic obstructive pulmonary disease. Thorax.

[27] Anthonisen NR, Lindgren PG, Tashkin DP, Kanner RE, Scanlon PD, Connett JE (2005). Bronchodilator response in the lung health study over 11 yrs. Eur Respir J.

[28] Vestbo J, Edwards LD, Scanlon PD, Yates JC, Agusti A, Bakke P (2011). Changes in forced expiratory volume in 1 second over time in COPD. N Engl J Med.

[29] Yoo KH (2015). Smoking cessation and chronic obstructive pulmonary disease. Korean J Intern Med.

