# Supplementary material for: Favorable longitudinal change of lung function in patients with asthma-COPD overlap from a COPD cohort
Source: Respir Res. 2018 Mar 2;19:36. doi: 10.1186/s12931-018-0737-8 (PMC5833074; doi:10.1186/s12931-018-0737-8)
Supplement: Supplementary file 1 — Table S1. The component distribution of ACO. Table S2. Longitudinal change of annual forced expiratory volume in 1 s (mL) in ACO by use of ICS/LABA or ICS during follow up (n = 47). (DOCX 15 kb) [file 12931_2018_737_MOESM1_ESM.docx]

**Table S1 The component distribution of ACO**

| Components | Overall  (n=239) | ACO  (n=47) |
| --- | --- | --- |
| History of bronchial asthma | 71 (29.7) | 39 (83.0) |
| Bronchodilator response to salbutamol/albuterol > 400 mL in FEV_1_ at baseline | 16 (6.7) | 13 (27.7) |
| History of atopy or allergic rhinitis | 29 (12.1) | 13 (27.7) |
| Two separated bronchodilator responses to salbutamol/albuterol higher than 12% and 200 mL in FEV_1_ during the initial 3-year follow-up period of the cohort | 56 (23.4) | 22 (46.8) |
| Peripheral blood eosinophils ≥ 300 cells/uL. | 76 (31.8) | 24 (51.1) |

Abbreviations: ACO, asthma-chronic obstructive pulmonary disease overlap

**Table S2 Longitudinal change of annual forced expiratory volume in 1 second (mL) in ACO by use of ICS/LABA or ICS during follow up (*n*=** **47)**

|  | ACO without ICS/LABA or ICS (n=13) | ACO with ICS/LABA or ICS (n=34) | *P for interaction†* |
| --- | --- | --- | --- |
| **Crude, *mL*** | -21.73 (-47.76, 4.30) | -10.44 (-26.34, 5.46) | 0.47 |
| **Model 1, *mL*** | -21.55 (-47.56, 4.46) | -10.23 (-26.12, 5.66) | 0.47 |
| **Model 2, *mL*** | -21.53 (-47.56, 4.50) | -10.22 (-26.12, 5.70) | 0.47 |

Data are presented as mean (95% confidence interval).

Abbreviations: ACO, asthma-chronic obstructive pulmonary disease overlap; ICS, inhaled corticosteroids; LABA, long-acting β2-agonists

*Defined when the ICS/LABA or ICS was prescribed longer than 2/3 of study periods.

Model 1: Adjusted for baseline age, baseline body mass index and smoking status during study period; Model 2: Further adjusted for at least 2 exacerbations per a year during study period;
